# Dental Caries and Associated Risk Indicators among Married Saudi Women

**DOI:** 10.1055/s-0041-1739437

**Published:** 2021-12-17

**Authors:** Syed Akhtar Hussain Bokhari, Kawthar Almumtin, Wala Mohammed Alhashiem, Duaa youssef Albandar, Zainab Nouh Alyahya, Ebtihal Alsaad

**Affiliations:** 1 Department of Dental Public Health, College of Dentistry, King Faisal University, Hofuf, Saudi Arabia; 2 Department of General Dentistry, Wroclaw Medical University, Saudi Arabia; 3 Department of General Dentistry, Farabi College, Saudi Arabia

**Keywords:** dental caries, decayed, missing, and filled teeth, married women, risk indicators

## Abstract

**Objective** The aim of this study was to evaluate decayed, missing, and filled teeth (DMFT) experience among married females in Saudi Arabia and provide an exploratory data for subsequent primary prevention.

**Materials and Methods** A cross-sectional quantitative study was conducted at a general hospital in Hofuf, Saudi Arabia. All married women attending the general hospital from March 1st to April 15 ^th^ , 2021 were requested to participate. Data was collected on a validated self-reported questionnaire consisting of sociodemographic factors, medical history, dietary pattern, and DMFT. Descriptive and regression analyses were performed using *p* ≤0.050.

**Results** Four hundred forty-eight married females with the mean age of 30.81 ± 6.11 years, mean duration of marriage of 9.55 ± 6.58 years, and having average number of children 2.32 ± 1.69 participated in the study. 61.7% mothers had ≥10 years of education. 63.6% were non-working and 56.5% were found with low family income. 66% participants reported of doing exercise less or more often yet 51.7% were ≥overweight. Consumption of energy drinks and dairy products was found significantly associated with increasing number of DMFT. Use of fluoridated toothpaste and dental visits was also found associated with increasing number of dental caries. Increasing age ( *p* = 0.040), increasing number of children, and middle family income were also significantly associated with higher DMFT, respectively ( *p* = 0.002, *p* = 0.022). In multi-logistic adjusted analysis, only consumption of dairy products, dental visits, and the unsure status of the use of fluoridated toothpaste were significantly associated with DMFT ≥1.

**Conclusion** DMFT status in married Saudi women was associated with participants' dietary habits, oral health-related practices, family income, married years, and number of children.

## Introduction

Dental caries, a multifactorial disease is recognized as a problem of public health significance with a high prevalence in adults. 1 The significant impact of caries on the world's population makes the disease an important topic of interest. 2 The burden of dental caries in children has equally been associated with the caries experience of mothers. The scores of decayed, missing, filled surfaces (DMFS) in mothers have been reported to have a direct significant correlation with the caries scores of their children. 3 Since women are also more likely to experience dental caries than men, and possessing a central position in family, mothers may be considered as targets for oral health promotion. 2 4

Studies have focused on the social determinants in the health and illness process and lifestyle has been associated with various diseases. 5 The individual level factors, health-related behaviors, and material factors play an important role. 6 Income is considered as a socioeconomic measure related to material conditions. Income affects eating patterns, housing, knowledge, and access to health care, all of them directly affect either exposure to risk or protection from disease. Education is also considered as an important component of socioeconomic status that contributes to health differences. 7 The dietary habits developed at a younger age are important as these behaviors are likely to remain stable for the entire lifespan. Adult food choices are not consistent with the dietary guidelines, leading to many preventable diseases. 8

Families are changing globally, including the Arabian region because of transitions in marriage, childbearing, fertility, lifestyle, increased participation of women in the labor force, educational achievements, cultural changes reflecting modernization, and a rapid pace of urbanization. Married women in Saudi Arabia make a reasonable size of the population. 9 The hypothesis for the present study was that severity of dental caries among adults is influenced by the demographic characteristics, oral health-related behavior as well as variables related to the dietary pattern. Thus, the aim was to assess the association between caries severity in married females and the characteristics of this population with respect to the different levels at which the determinants of caries operate (individual, socio-demographic and dietary pattern) and provide the required exploratory data for subsequent primary prevention.

## Materials and Methods

A cross-sectional quantitative study was conducted at Al-Maghlouth Hospital Hofuf, Al-Ahsa Saudi Arabia from March 1st to April 15 ^th^ , 2021. All married women attending the hospital were requested to participate. Data was collected on a validated questionnaire consisting of socio-demographic factors of age, education, occupation, family income, height and weight to calculate BMI, and exercise habit. Medical history was recorded for hypertension, cardiac disease, diabetes mellitus, and other chronic medical conditions. Dietary pattern included consumption of soft drinks, energy drinks, fast foods, dairy products, food supplement, meat products, eggs, vegetables, and number of meals per day. Participants were also inquired about oral hygiene practices consisting of mouth rinsing, use of toothbrush and frequency, toothpaste and visit to dentist. Dental status was recorded as decayed, missing, and filled teeth (DMFT). The outcome of interest was caries experience as determined by DMFT > 0: decayed tooth, missing, and filled tooth due to caries. The exposure variables were age, duration of marriage, number of children, family income, and educational qualification. Data obtained was entered and analyzed using Stata version 11.0. Descriptive and regression analysis was used to determine associations between variables and the cut off level of statistical significance set at 5% with 95% confidence interval. Study was approved by the ethical committee of the King Faisal University Saudi Arabia vide letter # KFU-REC-2020–11–25 dated November 30, 2020 and Al-Maghlouth hospital vide letter dated January 05, 2021.

## Results

### Socio-Demographic Characteristics

Six hundred thirty-five ( *n* = 635) married women visiting the Maglouth Hospital, Hofuf, Saudi Arabia were approached during the study period, 448 returned their completely filled self-reported questionnaire. The mean age of these women was 30.81 ± 6.11 years (range = 18–45 years), mean duration in marriage was 9.55 ± 6.58 years with a maximum duration of 30 years. On an average the number of children that these women bore was 2.32 ± 1.69 with a maximum number of eight children. Less than two-third (61.7%) of mothers were found with 10 years of education and above. Majority of them were non-working (63.6%) and more than half of them were found with low family income (56.5%). Around two-third of the participants reported of doing exercise less or more often (66%) yet 51.7% were found overweight or obese. Out of all participants, few were found suffering from hypertension (4.2%), diabetes mellitus (2.2%), cardiac disease (0.4%), and other chronic medical conditions (8%) ( Table 1 ).

**Table 1:** Demographic description of study participants ( *n* = 448)

Continuous variables		Mean ± SD	Range
Age (in years)		30.81 ± 6.11	18–45 y
Duration of marriage (in years)		9.55 ± 6.58	1–30 y
No. of family members		4.34 ± 1.73	1–10
No. of children		2.32 ± 1.69	0–8
Height (in cm)		158.72 ± 6.5	114–180 cm
Weight (in kg)		65.38 ± 13.44	30–116 kg
BMI (kg/m ^2^ )		25.95 ± 5.18	11.7–50.9
Categorical variables		Frequency (%)	
Educational level	No education	24 (5.4)	
	1–10 y	148 (33)	
	>10–14 y	222 (49.6)	
	>14 y	54 (12.1)	
Occupation	Non-working	285 (63.6)	
	Business/Self employed	40 (8.9)	
	Employed	123 (27.5)	
Family income	≤10,000	253 (56.5)	
	>10,000–20,000	132 (29.5)	
	>20,000	63 (14)	
BMI	Underweight	22 (4.9)	
	Normal weight	193 (42.9)	
	Overweight	137 (30.4)	
	Obese	96 (21.3)	
Exercise	Never	149 (33.3)	
	Less often	207 (46.2)	
	More often	92 (20.5)	
Hypertension	Yes	19 (4.2)	
	No/Don't know	429 (92.1)	
Cardiac disease	Yes	2 (0.4)	
	No/Don't know	446 (99.6)	
Diabetes	Yes	10 (2.2)	
	No/Don't know	438 (97.3)	
Any other condition	Yes	37 (8.0)	
	No/Don't know	411 (92)	

### Dietary Pattern

Table 2 gives details of participants' dietary pattern. Majority of the study participants reported of consuming some unhealthy food items such as fast food (80.4%) and soft drinks (64.7%). On the other hand, there were majority who reported of consuming healthy food items also such as fruits (96%), dairy products (91%), eggs (92.6%), meat products (95.3%), and vegetables (97.5%). Comparatively, less consumed items were energy drinks (14.7%) and food supplements (45.1%). Majority preferred taking meals less than three times per day (83.4%).

**Table 2:** Description of participants' dietary intake ( *n* = 448)

Food items		Frequency (%)
Soft drink	Yes	290 (64.7)
	No	158 (35.3)
Energy drinks	Yes	66 (14.7)
	No	382 (85.3)
Fast food	Yes	360 (80.4)
	No	88 (19.6)
Fruits	Yes	430 (96)
	No	18 (4)
Dairy products	Yes	409 (91.3)
	No	39 (8.7)
Food supplements	Yes	202 (45.1)
	No	246 (54.9)
Eggs	Yes	415 (92.6)
	No	33 (7.4)
Meat products	Yes	427 (95.3)
	No	21 (4.7)
Vegetables	Yes	437 (97.5)
	No	11 (2.5)
No. of meals/d	<3 times	374 (83.5)
	3 times	72 (16.1)
	>3 times	2 (0.4)

### Oral Hygiene Practices

Table 3 explains the participants' oral health practices. About 90% reported for rinsing mouth after meals. More than half of the study participants were in a habit of brushing teeth for more than once a day (twice daily = 38.4%, thrice daily = 12.5%). Miswak was seldom used by these study participants for cleaning their teeth (11.4%, similarly dental floss (28.1%). One-third of the participants reported of using fluoridated toothpaste (66.1%) for brushing their teeth. Only less than 5% of the participants reported that they have never changed their brush (4.5%) and 10% reported that they have never visited any dentist.

**Table 3:** Description of self-reported oral health practices and dental caries status of study participants ( *n* = 448)

Oral health-related questionnaire	Responses	Frequency (%)	
Do you rinse after meals?	Don't rinse	48 (10.7)	
	Once daily	119 (26.6)	
	Twice daily	123 (27.5)	
	Thrice daily	158 (35.3)	
How many times do you brush your teeth?	Don't brush	38 (8.5)	
	Once daily	182 (40.6)	
	Twice daily	172 (38.4)	
	Thrice daily	56 (12.5)	
Do you use miswak to brush your teeth?	Yes	51 (11.4)	
	No	397 (88.6)	
Do you floss your teeth?	Yes	126 (28.1)	
	No	291 (65)	
	Don't know	31 (6.9)	
Do you use fluoridated toothpaste?	Yes	296 (66.1)	
	No	108 (24.1)	
	Don't know	44 (9.8)	
When do you change your brush?	Never changed	20 (4.5)	
	Every 3 mo	163 (36.4)	
	Every 6 mo	112 (25)	
	As needed	153 (34.2)	
When do you visit dentist?	Never visited	45 (10)	
	Every 6 mo	59 (13.2)	
	As needed	344 (76.8)	
**Dental caries status**	**No. of teeth**	**Frequency (%)**	**Mean ± SD**
Decayed teeth	0–13	58.04	1.38 ± 1.67
Filled teeth	0–16	73.67	2.14 ± 2.24
Missed teeth	0–9	42.86	0.82 ± 1.34
DMFT	0–22	88.39	4.34 ± 3.46

Abbreviation: DMFT, decayed, missing and filled teeth.

### Decayed, Missing, and Filled Teeth Status

The dental caries status of these women showed that on an average these women had 1.38 ± 1.67 decayed (D), 0.81 ± 1.34 missing due to caries (M), and 2.14 ± 2.24 filled (F) teeth. Their mean DMFT was calculated as 4.34 ± 3.46 with a range between 0 and 22 teeth suffering from dental caries. Out of total study participants only 11.61% ( *n* = 52) were found caries free (DMFT = 0). More than half (58.04%) of the study participants were found with decayed, 42.86% having more than one missing teeth due to caries and 73.66% having more than one filled teeth. Maximum number (14.96%, *n* = 67) of participants were found to have DMFT = 4. Only 27 (6.02%) participants were found to have DMFT >10.

### Association of Demographic Variables and DMFT

Table 4 shows crude association of demographic variables of the study participants with dental caries status considered as DMFT = 0 and DMFT ≥1. This table shows that age group 26 to 35 years were significantly found associated with increasing number of DMFT scores. Similarly, participants belonging to age group 35 and above were also significantly associated ( *p* = 0.004) with increasing DMFT score. Increasing number of children of the Saudi married women and middle family income were also significantly associated with increasing number of DMFT [ *p* = 0.002, *p* = 0.022]. There was no association between DMFT and BMI ( *p* > 0.05).

**Table 4:** Crude association between demographic variables and dental caries status (mean DMFT = 0; ≥1) among all study participants ( *n* = 448)

Demographic variables		*n* (%)	Unadj. OR [95% CI]	*p* -Value
Age groups				
	18–25 y	83 (18.53)	Ref.	Ref.
	26–35 y	242 (54.02)	5.80 [2.852, 11.799]	**<0.001**
	35–45 y	123 (27.46)	2.98 [1.430, 6.226]	**0.004**
Years in marriage				
	≤5 y	153 (34.15)	Ref.	Ref.
	>5 y	295 (65.85)	1.77 [0.989, 3.182]	0.054
No. of children				
	No children	70 (15.63)	Ref.	Ref.
	1–2 children	194 (43.30)	3.13 [1.510, 6.511]	**0.002**
	>2 children	184 (41.07)	3.15 [1.503, 6.603]	**0.002**
Educational level				
	No education	23 (5.15)	Ref.	Ref.
	Secondary	148 (33.11)	0.54 [0.119, 2.492]	0.434
	Undergraduate	222 (49.66)	0.78 [0.173, 3.560]	0.754
	Postgraduate	54 (12.08)	0.95 [0.160, 5.634]	0.957
Family income in Saudi Rials				
	≤10,000	253 (56.47)	Ref.	Ref.
	>10,000–20,000	132 (29.46)	2.67 [1.151, 6.233]	**0.022**
	>20,000	63 (14.06)	0.63 [0.307, 1.319]	0.225
BMI				
	Normal weight	193 (42.08)	Ref.	Ref.
	Overweight	137 (30.58)	1.68 [0.842, 3.378]	0.140
	Obese	96 (21.43)	1.94 [0.852, 4.436]	0.114
	Underweight	22 (4.91)	1.76 [0.392, 7.974]	0.458

Note: *p* -Values set in bold are significant.

### Association of Dietary Variables, Oral Hygiene Practices, and DMFT

Crude analyses of self-reported daily dietary intake and dental hygiene practices on DMFT status also show that consumption of energy drinks ( *p* = 0.029) and dairy products ( *p* = 0.028) was found to be significantly associated with increasing number of DMFT. Use of fluoridated toothpaste ( *p* = 0.041) and dental visits ( *p* = 0.014) was also found associated with increasing number of dental caries ( Table 5 ). When all significant variables were run through multi-logistic adjusted analysis it was observed that only the consumption of dairy products ( *p* = 0.020), dental visits ( *p* < 0.001) and the unsure status of use of fluoridated toothpaste ( *p* = 0.021) were significantly associated with DMFT ≥1 ( Table 6 ).

**Table 5:** Crude association of participants' dietary intake and teeth cleaning practices with respect to dental caries status (mean DMFT = 0; ≥1) among all study participants ( *n* = 448)

Variables	Categories	*n* (%)	Unadj. OR [95% CI]	*p* -Value
Soft drink	No	158 (35.27)	Ref.	Ref.
	Yes	290 (64.73)	1.40 [0.778, 2.523]	0.260
Energy drinks	No	382 (85.27)	Ref.	Ref.
	Yes	66 (14.73)	**0.46 [0.232, 0.925]**	**0.029**
Fast food	No	89 (19.87)	Ref.	Ref.
	Yes	359 (80.13)	1.57 [0.813, 3.057]	0.178
Fruits	No	19 (4.24)	Ref.	Ref.
	Yes	429 (95.76)	0.89 [0.200, 3.974]	0.881
Dairy products	No	40 (8.93)	Ref.	Ref.
	Yes	408 (91.07)	**2.46 [1.100, 5.520]**	**0.028**
Food supplements	No	247 (55.13)	Ref.	Ref.
	Yes	201 (44.87)	0.94 [0.527, 1.684]	0.843
Eggs	No	36 (8.04)	Ref.	Ref.
	Yes	412 (91.96)	0.67 [0.199, 2.278]	0.525
Meat products	No	23 (5.13)	Ref.	Ref.
	Yes	425 (94.87)	0.33 [0.043, 2.526]	0.288
Vegetables	No	16 (3.57)	Ref.	Ref.
	Yes	432 (96.43)	0.49 [0.064, 3.850]	0.504
No. of meals/day	≤3 times	374 (83.48)	Ref.	Ref.
	>3 times	74 (16.52)	1.30 [0.566, 3.028]	0.529
Flossing	No	322 (71.88)	Ref.	Ref.
	Yes	126 (28.13)	1.19 [0.616, 2.328]	0.594
Fluoride toothpaste	No	107 (23.88)	Ref.	Ref.
	Yes	296 (66.07)	**1.91 [1.026, 3.569]**	**0.041**
	Not sure	45 (10.04)	3.02 [0.847, 10,784]	0.088
Rinse after meals	Don't rinse	48 (10.71)	Ref.	Ref.
	Once daily	119 (26.56)	1.26 [0.409, 3.923]	0.681
	Twice daily	123 (27.46)	0.77 [0.268, 2.255]	0.643
	Not sure	158 (35.27)	0.75 [0.269, 2.132]	0.600
Brushing frequency	Don't brush	38 (8.48)	Ref.	Ref.
	Once daily	182 (40.63)	1.94 [0.707, 5.350]	0.197
	Twice daily	172 (38.39)	1.50 [0.558, 4.078]	0.416
	Not sure	56 (12.50)	0.76 [0.257, 2.288]	0.634
Changing brush	Never changed	20 (4.46)	Ref.	Ref.
	Every 3 mo	163 (36.38)	1.60 [0.490, 5.236]	0.435
	Every 6 mo	112 (25)	2.86 [0.787, 10.395]	0.110
	Not sure	153 (34.15)	2 [0.598, 6.680]	0.260
Dental visit	Never visited	57 (12.72)	Ref.	Ref.
	Every 6 mo	59 (13.17)	**3.18 [1.261, 8.052]**	**0.014**
	As needed	332 (74.11)	**6.14 [3.094, 12.182]**	**<0.001**

Note: *p* -Values set in bold are significant.

**Table 6:** Multiple logistic regressions

DMFT = 0 v/s ≥1	Adj. ORs [95% CI]	*p* -Value
Age	1.05 [0.990, 1.134]	0.092
No. of children	0.94 [0.741, 1.194]	0.620
Family income	0.82 [0.541, 1.267]	0.387
Consume energy drinks	0.64 [0.288, 1.424]	0.27
Consume dairy products	2.83 [1.175, 6.834]	**0.020**
Use fluoridated toothpaste (yes)	1.60 [0.821, 3.139]	0.166
Use fluoridated toothpaste (no sure)	5.11 [1.278, 20.463]	**0.021**
Dental visits	2.60 [1.790, 3.795]	**<0.001**

Note: LR Chi-square = 42.34 [ *p* < 0.001] _cons = 0.14 [0.018, 1.188] *p* = 0.072. *p* -Values set in bold are significant.

## Discussion

Motherhood age is influenced by complex socioeconomic, educational, and cultural factors, which differ significantly for different communities. 10 Worldwide, the prevalence of dental caries among adults is high as the disease affects nearly 100% of the populations in the majority of countries. 11 Pregnancies have several negative effects on the oral cavity environment, a compelling reasoning why women have greater caries activity than men. 2 This first study on married women from Saudi Arabia has explored dental caries experience and associated risk indicators. Demographic data of this study with mean age of 30.8 years, 76% of education, average family size of 4.34 persons, average number of children 2.3, 37% having some occupation, and 44% house hold income for Saudi women are close to and comparable with a latest study from Saudi Arabia. 9 In this study, nearly half of the participants (50.7%) were between 21 and 30 years, only 3.6% were below 20 years and 0.7% over 40 years. The majority of the participants were housewives; with comparable proportions between the different age groups. 12% of women achieved university or higher education. The mean BMI of the participants showed a trend of increasing proportions through the age groups from as low as 6.2% obese mothers among less than 20 years to as high as 33.3% among mothers >40 years of age and these values are very much comparable with those of the RAHMA study from Riyadh. 10

Oral health of Saudi Arabian population has been reported to be influenced by several socio-demographic factors as well by improper oral hygiene practices, limited use of preventive dental services, and low percentage of population seeking routine dental check-up despite having free access to dental care has pointed toward the lack of awareness about oral health. Improvement in the education of women influences the duration of marriage, the ideal number of children, age of women at delivery of their last child. 12

The study has found prevalence of dental caries among 88% of participants as compared with 25.3% of women of another study 1 and 63.2% of 19 to 21 years old females in an Indian study with and average DMFT of 3.26. 13 This prevalence is very much coherent with dental caries experience reported among female populations from Korea (91.6%) 14 and Spain (93.3%). 11 The mean DMFT score among the women in this study was 4.34. DT, MT, FT components respectively were 58.04, 42.86, 73.64% that is very low in comparison with mean DMFT of 15.5 ± 4.5, of another study. 15 But this study sample showed a moderately severe dental caries experience as compared with 89% of sample categorized in the “Extremely High” dental caries experience by other study. 15 A greater prevalence of high caries severity was found among those who frequently visited the dentist, 5 a finding similar to this study.

It is recorded that women who gave birth to more children show a higher percentage of “decays” compared with women with only one child 16 ; this study has also shown that caries was associated with number of children. Studies in other countries have reported mean DMFT scores ranging from 3.09 to 7.89 among women of similar age to our study population. 11 17 The values obtained in a study were DMFT (7.89), DT (0.64), MT (1.95), and FT (5.31) in the 35- to 44-year-old female group. 11

Furthermore, similar to this study, another study 18 also reported a mean DMFT of 3.88 among women in a hospital-based study. Although males were not observed in this study; however, study has reported that dental caries rate and tooth loss are higher in females than men and more often result from dental caries. 19 This study population has shown low-severity level of dental caries, while using another definition of the severity of dental caries: DMFT≥ 14 as high severity category, and DMFT < 14 as low severity. 20 In this study, caries severity remained significantly associated with age, regular dentist visit, and household income as reported elsewhere. 5

Lack of oral hygiene and its ill-effects on oral health can be avoided by good oral hygiene practices. 21 Oral hygiene of women of this study has been observed at a level that is comparable with other studies. 19 20 21 22 In this study, tooth brushing frequency was 40.6% once daily, 38.4% twice daily, and 12.5% thrice comparable with that of 24.9% once per day, 38.5% twice per day, 36.3% three times per day. Chewing stick users were at 12.2% compared with this study that was 11.4%. 23 Oral health practices may be improved by enhancing awareness through transmitting knowledge that leads to positive attitude and good health-related behaviors. 12

A dramatic lifestyle change is noticed in Saudi population over the last few decades; this change is not only in the form of sedentary lifestyle but also in the dietary patterns. 22 Dietary habits can have a major impact based on the form and frequency of the food. Particularly dietary routines have been shown to increase the incidence of caries 2 Fast food consumption frequency (80%) by our study participants was higher compared to a study conducted in Riyadh where it was approximately 75%. 23 Fast food was consumed once per week by 52.8% of adolescent girls and 60.9% of young adult girls (19–29 years). A large majority of women in a comparative study community had never visited a dentist or received any dental care, comparable to this study where 90% visited dentist. 24

## Conclusion

In this study, almost all women had good oral hygiene practices and were engaged in tooth cleaning procedures. Self-reported status of DMFT was significantly associated with increasing age, number of children, and moderate family income. Multiple logistic regression exhibited significant associations of DMFT with the consumption of dairy products, fluoridated toothpaste, and dental visits. These findings highlight the challenges to dental health practice, particularly the importance of risk assessment in estimating the potential for prevention.
